# *ELFN1-AS1*: A Novel Primate Gene with Possible MicroRNA Function Expressed Predominantly in Human Tumors

**DOI:** 10.1155/2014/398097

**Published:** 2014-02-24

**Authors:** Dmitrii E. Polev, Iuliia K. Karnaukhova, Larisa L. Krukovskaya, Andrei P. Kozlov

**Affiliations:** ^1^Biomedical Center, 8 Vyborgskaja Street, Saint Petersburg 194044, Russia; ^2^Department of Genetics and Breeding, Saint Petersburg State University, 7/9 Universitetskaya Embankment, Saint Petersburg 199034, Russia

## Abstract

Human gene *LOC100505644 uncharacterized LOC100505644 [Homo sapiens]* (Entrez Gene ID 100505644) is abundantly expressed in tumors but weakly expressed in few normal tissues. Till now the function of this gene remains unknown. Here we identified the chromosomal borders of the transcribed region and the major splice form of the *LOC100505644*-specific transcript. We characterised the major regulatory motifs of the gene and its splice sites. Analysis of the secondary structure of the major transcript variant revealed a hairpin-like structure characteristic for precursor microRNAs. Comparative genomic analysis of the locus showed that it originated in primates *de novo*. Taken together, our data indicate that human gene *LOC100505644* encodes some non-protein coding RNA, likely a microRNA. It was assigned a gene symbol *ELFN1-AS1* (ELFN1 antisense RNA 1 (non-protein coding)). This gene combines features of evolutionary novelty and predominant expression in tumors.

## 1. Introduction

UniGene cluster Hs.633957 (Ref: UGID:2140851) corresponds to the human gene *LOC100505644 uncharacterized LOC100505644 [Homo sapiens]* (Entrez Gene ID: 100505644), which resides in the 7th chromosome in the intron of *ELFN1* gene. It was found to be expressed primarily in human tumors by *in silico* analysis [1] and by experimental detection of *LOC100505644 *transcripts in tumors of various histological origin. In general, transcripts were absent from most normal tissues, but some weak expression signals could be detected in adult heart and digestive system [2–4].

The broad spectrum of positive tumors and only few positive normal tissues make this gene potentially meaningful for oncology; however, the question whether the *LOC100505644* transcripts are functional or just a tumor-related transcriptional noise remained unanswered. Here we confirm the tumor-associated expression pattern of the gene using an independent set of samples and present experimental and *in silico* evidence that *LOC100505644* shows alternative splicing, possesses its own promoter, and is likely to code for a miRNA, which is the evidence of a functional gene. Interestingly, this gene seems to have originated in primates *de novo* from an intronic region of the *ELFN1* gene. It was assigned a gene symbol *ELFN1-AS1 *(ELFN1 antisense RNA 1 (non-protein coding)).

## 2. Material and Methods

### 2.1. RNA and cDNA

Total RNA samples from human placenta and ovary carcinoma were obtained from Ambion, TX, USA. Total RNA samples from human moderately differentiated endometrium adenocarcinoma and lung squamous cell carcinoma were kindly provided by Dr. Kolyubaeva, Kirov Military Medical Academy, St. Petersburg, Russia. Appropriate informed consent was obtained. cDNA preparations from human healthy tissues were purchased from Clontech, CA, USA (Human MTC Panels). Human tumor cDNA preparations were purchased from BioChain, CA, USA (catalog nos.: C8235544, C8235545, C8235546, C8235547, and C8235549).

### 2.2. PCR

The quality of cDNA panels was assessed by PCR with primers for the *GAPDH* gene (forward 5′-TGAAGGTCGGAGTCAACGGATTTGGT-3′ and reverse 5′-CATGTGGGCCATGAGGTCCACCAC-3′). Annealing temperature was 68°C and amplicon size was 983 bp. Twenty-five cycles of PCR were performed. Gene-specific primers were as follows: 5′-GGTCTTTACTCCCATTCAA-3′ and 5′-CTCCTGTCATTCACTCCG-3′. The reaction was conducted in 25 *μ*L of a solution containing 2.5 ng of cDNA, PCR buffer (67 mM Tris HCl, pH 8.9, 4 mM MgCl_2_, 16 mM (NH_4_)_2_SO_4_, 10 mM 2-mercaptoethanol, and 0.1 mg/mL BSA), dNTPs (200 *μ*M each), 0.4 *μ*M each primer, and 1 U of Taq DNA polymerase. PCR program: denaturation at 95°C for 1 min followed by 35 cycles: 95°C for 30 s, 56°C for 30 s, and 72°C for 30 s. Postextension was carried out at 72°C for 5 min. The resulting amplificates were resolved by electrophoresis in 2% agarose gel and stained with ethidium bromide. The presence of the mRNA under study in the sample was indicated by the presence of the 322 bp long amplification product. The gels were photographed under UV illumination.

### 2.3. RACE

We used SMART RACE cDNA Amplification Kit (Clontech) with ovary carcinoma and placenta RNA samples and Marathon cDNA Amplification Kit (Clontech) with uterus adenocarcinoma and lung carcinoma RNA samples for RACE experiments. cDNA was prepared as recommended by the manufacturer. Gene-specific primers 5gsp1, 5gsp1n, 5gsp2, 5gsp2n, 5gsp3, 5gsp3n, 3gsp1, and 3gsp1n for the amplification of the 5′- and 3′-cDNA ends are presented in Table 1.

cDNA ends were amplified using Advantage 2 PCR Kit (Clontech) with gene-specific and Marathon adaptor primers under the following conditions: 1 cycle of 94°C for 2 min, 5 cycles of 94°C for 30 sec, 72°C for 3 min, 5 cycles of 94°C for 30 sec, 70°C for 3 min, 35 cycles of 94°C for 30 sec, 68°C for 3 min, and 1 cycle of 68°C for 5 min. A 100-fold diluted 1 mkl aliquote from the 1st round of amplification was used for the 2nd round of amplification with nested primers under the same conditions. PCR products were further cloned into the pGEM-T Easy Vector (Promega, WI, USA), propagated in *E. coli,* and sequenced using conventional techniques.

Primers for identification of the major splice variant are presented in Table 1. Amplifications were performed under the following conditions: 1 cycle of 95°C for 2 min, 15 cycles of 95°C for 30 sec, 58°C for 30 sec, and 72°C for 1 min and 1 cycle of 72°C for 5 min. A 1 mkl aliquote from the 1st round of amplification was used for the 2nd round of amplification with nested primers. Cycling conditions were as above, but 35 cycles of amplification were used.

### 2.4. Software and Databases

We used BioEdit software [5] for basic manipulations with nucleic and amino acids sequences. Resources of the NCBI databases (http://www.ncbi.nlm.nih.gov/) and UCSC Genome Browser (GB) [6, 7] (http://genome.ucsc.edu/) were used extensively. RNA folding was done using Mfold [8] (http://www.bioinfo.rpi.edu/applications/mfold/) and Vienna RNA Websuite [9] online software (http://rna.tbi.univie.ac.at/cgi-bin/RNAfold.cgi) with default settings. PROMO3 web software [10] (http://alggen.lsi.upc.es/cgi-bin/promo_v3/promo/promoinit.cgi?dirDB=TF_8.3) was used for identification of putative TFBS. NetGene2 Server [11] (http://www.cbs.dtu.dk/services/NetGene2/) was used for identification of potential splice sites in nucleotide sequences.

### 2.5. Genomic Sequences

We used the following genomic sequences for the comparative analysis: *Otolemur garnettii* (GI:393210271, positions: 4461840-4466746), *Callithrix jacchus* (GI: 290467407, positions: 56153639-56159372), *Saimiri boliviensis* (GI: 395721681, positions: 473433-479005), *Macaca mulatta* (GI: 109156890, positions: 39676154-39682121), *Papio anubis* (GI: 395728659, positions: 34224856-34230574), *Nomascus leucogenys* (GI: 328833306, positions: 1257019-1262659), *Pongo abelii* (GI: 241864935, positions: 1664355-1670047), *Pan paniscus* (GI: 393728162, positions: 1586174-1591896), *Pan troglodytes* (GI: 319999821, positions:417230-422955), *Homo sapiens* (hg19, chr7: 1777236-1782938), and *Gorilla gorilla* (gorGor3.1/gorGor3, chr7: 1,684,515-1,691,410, gaps excluded).

## 3. Results

### 3.1. Expression of the Gene in Human Normal Tissues and Tumors

The specificity of expression of our gene was studied using PCR with gene-specific primers and panels from normal and tumor tissues.

**Figure 1:**
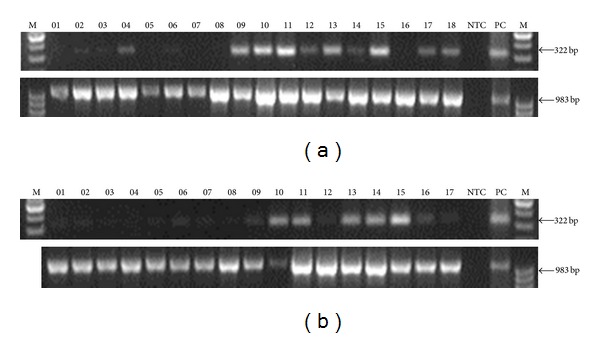
Expression of the *ELFN1-AS1 *gene in human normal tissues and tumors. (a) Upper pane: PCR with *ELFN1-AS1*-specific primers. (a) Lower pane: *GAPDH* control. (a) Lanes: M: DNA size marker; 1: normal brain; 2: normal heart; 3: normal kidney; 4: normal liver; 5: normal lung; 6: normal pancreas; 7: normal placenta; 8: normal skeletal muscle; 9: brain, malignant meningioma, moderately differentiated; 10: lung, nonsmall cell carcinoma; 11: kidney, transitional cell carcinoma, papillary moderately differentiated; 12: kidney, renal cell carcinoma; 13: liver, hepatocellular carcinoma, well differentiated; 14: liver, adenocarcinoma, moderately differentiated; 15: gallbladder, adenocarcinoma; 16: esophagus, squamous cell carcinoma, ulcer, well differentiated; 17: stomach, adenocarcinoma, ulcer, well differentiated; 18: small intestine, mesenchymoma, moderately differentiated; NTC: no template control; PC: positive control. (b) Upper pane: PCR with *ELFN1-AS1*-specific primers. (b) Lower pane: *GAPDH* control. (b) Lanes: M: DNA size marker; 1: normal colon; 2: normal ovary; 3: normal peripheral blood leukocytes; 4: normal prostate; 5: normal small intestine; 6: normal spleen; 7: normal testis; 8: normal thymus; 9: colon, adenocarcinoma, moderately differentiated; 10: rectum, adenocarcinoma, moderately/poorly differentiated; 11: ovary, adenocarcinoma, ulcer, moderately differentiated; 12: fallopian tube, medullary carcinoma, poorly differentiated; 13: uterus, adenocarcinoma; 14: ureter, moderately differentiated, transitional cell carcinoma; 15: bladder, transitional cell carcinoma, papillary; 16: testis, germinoma; 17: parotid, squamous cell carcinoma, mixed; NTC: no template control; PC: positive control.

The results are presented in Figure 1. They are in agreement with our previous data and show that *ELFN1-AS1* is more abundant in tumors, with week expression in normal tissues, for example, in liver (Figure 1(a), 04) and in heart (Figure 1(a), 02).

### 3.2. Primary Structure of the Hs.633957-Specific Transcript

To identify the borders of the transcribed region we conducted a series of 5′- and 3′-RACE experiments using RNA from lung, uterus, and ovarian tumors and human normal placenta. We obtained 7 different RACE ragments; 3 of them corresponded to the spliced 5′-end of RNA (GenBank accession nos. HO663743, HO663744, HO663747), the other 3 corresponded to the unspliced 5′-end of RNA (GenBank accession nos. HO663745, HO663746, HO663748) and 1 to the 3′-end of the RNA (GenBank accession no. HO663742).

**Figure 2:**
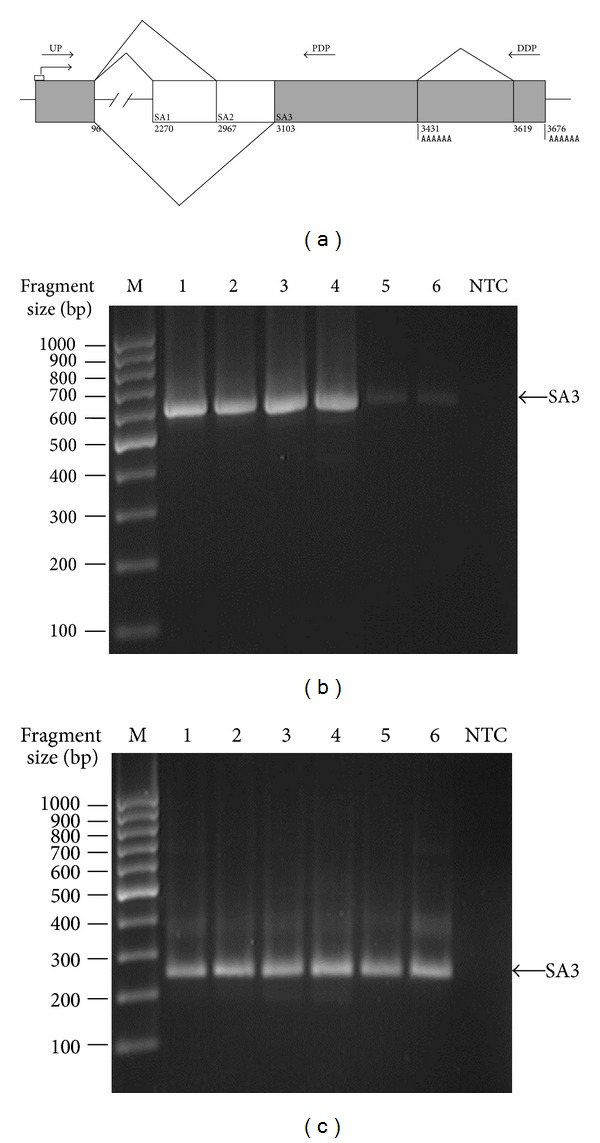
Identification of the primary structure of the Hs.633957-specific RNA. (a) Scheme of the transcript variants for the locus Hs.633957. Splice sites and polyadenylation sites are marked and their positions are given according to the leftmost TSS. Arrows indicate position of the primers which were used for identification of the major splice form. BX119057-like variant is filled in dark grey. (b) Results of cDNA PCR amplification with primers bordering the 39 to 3642 gene region. (c) Results of cDNA PCR amplification with primers bordering the 39–3289 gene region. cDNA samples: 1: gallbladder, adenocarcinoma; 2: rectum, well differentiated adenocarcinoma; 3: ureter, papillary transitional cell carcinoma; 4: T-cell Hodgkin's lymphoma; 5: kidney; 6: liver; NTC: no template control.

Based on the alignment of the Hs.633957-specific ESTs with the human genome (human genome assembly NCBI36/hg18), 3 alternative splice acceptor sites (SA) and 2 polyadenylation sites could be predicted for the *ELFN1-AS1 *RNA (Figure 2(a)). To identify the major transcript splice form we conducted nested PCR with the primers UP × DDP and nested primers NUP × NDDP (position from 39 to 3642), which border the transcribed region (Figure 2(a)), on the cDNA template from various tumors with known locus expression. Only the products with distal polyadenilation sites could be detected (Figure 2(b)). In each case a major amplicon corresponding to the transcript with the 1st intron splicing out at SA3 site was observed. To verify this result we conducted nested PCR with primers UP × PDP and nested primers NUP × NPDP specific for a shorter region of the gene (positions 39-3289) on the template of the same cDNA samples. The selected position of the primers allowed amplification of cDNAs corresponding to the RNAs with both distal and proximal polyadenilation sites. Again, the major amplicon corresponded to the transcript with splicing at SA3 (Figure 2(c)). Faint signals for amplicons about 380 bp might correspond to the transcript with splicing at SA2 (expected size 378 bp); however, no validation was performed. Thus, SA3 was the major splice acceptor site of the 1st intron.

**Figure 3:**
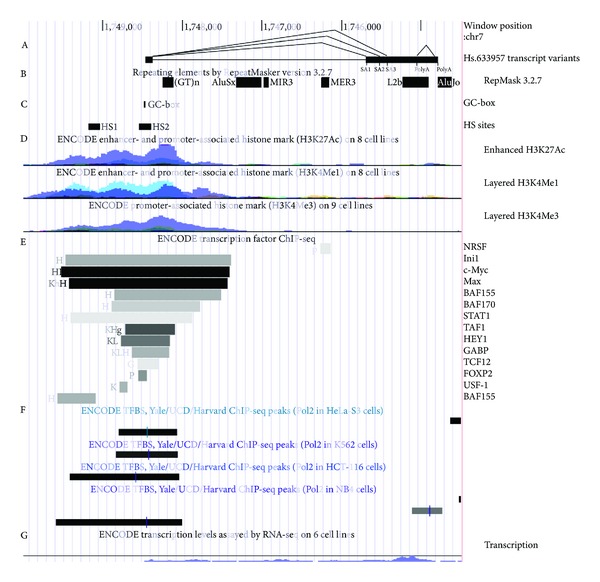
Transcribed locus Hs.633957 overview. A sketch of the chromosome 7 region containing transcribed locus Hs.633957 is shown according to the human genome assembly NCBI35/hg18. (A) Transcript variants are given to show the location of the region. (B) Repeating elements by RepeatMasker version 3.2.7 track indicates the location of repeating elements. (C) GC-box marks the GC-box motif identified in current study. HS sites mark position of the DNase I hypersensitivity sites. Location of the sites was estimated as averages of the left and right peak boundaries coordinates among the overlapping peaks from independent experiments for which data is available from UCSC Genome Browser (24 for HS1 and 17 for HS2). (D) Enhanced H3K27Ac, H3K4Me1, and Promoter H3K4Me3 tracks indicate association of the specific histone marks with the genome in certain cell lines. Overlaid data for several cell lines is shown. (E) ENCODE transcription factor ChIP-seq track provides a summary for association of various TFs with chromatin in various cell lines. Cell lines are indicated by letter code (H: HeLa-S3, K: K-562, h: HUVEC, g: GM12891, L: HepG2, G: GM12878). (F) Association of Pol 2 with chromatin in various cell lines is shown according to ENCODE ChIP-seq data. (G) ENCODE data on transcription levels in various cell lines are obtained by RNA-seq. Overlaid data is shown.

We used RepeatMasker ver. 3.2.7 integrated into the GB and identified several repeat units within the gene: SINEs, LINE, simple (TG)*n* DNA repeat, and a DNA transposon (Figure 3, B). However, the LINE L2b repeat was not recognized by the more recent RepeatMasker version open-3.3.0. The (TG)*n* repeat is of particular interest. It is located 129 bp downstream from the splice donor site (SD) (CAAG^∧^GTAA) of the 1st intron in the positive DNA strand. It is 136 bp long and holds 33 TG dinucleotides. Its divergence from the consensus repeat sequence is 36%.

### 3.3. Promoter Region of the Putative Gene

We used “ENCODE Enhancer and Promoter Histone Marks” and “ENCODE Transcription Factor ChIP-seq” tracks of the GB to identify the promoter region of the putative gene and detected promoter and enhancer epigenetic marks within the −1000 to +500 bp region, relative to the TSS, as well as association of various TFs with this region (Figure 3). We searched for the most common eukaryotic core promoter motifs, such as TATA-box, CCAAT-box, and GC-box within the region and verified them using the Bucher's position-specific weight matrices [12]. A GC-box located at the position from −13 to +1 satisfied the selection criteria (Figure 3(C); Supplementary File 1 avaliable online at http://dx.doi.org/10.1155/2014/398097).

We analysed the data available from the “ENCODE Univ. Washington DNaseI Hypersensitivity by Digital DNaseI” track of the GB and noticed that peaks of DNaseI hypersensitivity signal in various cell lines were concentrated around two sites (“HS1” and “HS2”) within the region under study (Figure 3(C)). The GC-box coincided with the center of HS2 site (Figure 3, “HS2” and “GC-box”), which supports the idea that this GC-box is an active promoter element.

Analysis of ChIP-seq data from various GB tracks revealed association of Ini1, c-Myc, Max, BAF155, BAF170, STAT1, TAF1, HEY, GABP, TCF12, FOXP2, USF1, SETDB1, PolII, E2F-1, E2F-4, E2F-6, YY1, and GATA-2 with the chromatin region from −1000 to +500 in certain cell types (Figure 3(E); Supplementary Figures S1–S5). Association of c-Myc and Max with the chromatin region was of particular interest, since the binding signal was the highest for these proteins, as indicated by the darkest color in Figure 3. We searched for canonical and noncanonical c-Myc-binding sites [13] (E-boxes) within the chromosome region and identified a CACGTG “E-box 1” and a CGCGTG “E-box 2” motifs 280 bp and 75 bp upstream of the TSS, respectively, (Supplementary Figure S1; Supplementary File 1). However, the most intriguing was the 85 bp region located 210 bp downstream of the gene's TSS, because it contained 6 Myc/Max-binding motifs (“E-boxes” 3–8, Supplementary Figure S1). Matching the chromosomal location of these Myc/Max sites with all the ChIP-seq data for Myc/Max available from GB revealed that c-Myc and Max bind the whole region surrounding the TSS, with the highest signal rate around the E-boxes 2–8 (Supplementary Figure S1).

For the other E-box-binding proteins, USF1 binding signal overlapped with the E-boxes 1–8 (Supplementary Figure S1). Transcription factors TCF12 and HEY1 were associated with the TSS, rather than with the E-boxes (Supplementary Figure S1).

We analysed the −1000–+500 region of the gene for the presence of putative TFBS using PROMO3 web software, which utilizes TRANSFAC ver. 8.3 TFBS matrixes. Of all the TFs, which were associated with the promoter proximal region of the gene, E2Fs, YY1, GABP, and GATA2 are known to bind specific DNA motifs other than E-boxes. Using PROMO3 we identified multiple putative TFBS for E2F and YY1 proteins within the chromatin region associated with these proteins (Supplementary Figures S2-S3; Supplementary File 1). A single GABP-binding site was identified at position from −17 to −6 (Supplementary File 1) within the chromosome region associated with GABP in HeLa-S3, HepG2 and K-562 cells (Supplementary Figure S4). Using PROMO3 we failed to identify any GATA2-binding sites within the region under study; however, there was a GATA1-binding site there according to the “HMR Conserved Transcription Factor Binding Sites” track, which holds location and score for the transcription factor binding sites conserved in the human/mouse/rat alignment. This site coincides with the peak point of GATA-2-binding in K-562 cells (Supplementary Figure S5).

### 3.4. Analysis of the ORFs

We identified two ORFs coding for 53 and 62 a.a. proteins. Amino acid sequence of the first ORF was MLELLLPLQEQELCVLVTAVASQGRRGAQQPGLDRLGPRCARAGMRLLCFLFR and that of the second ORF was MGLRSFSLPVLWCMPPSWLKNLHQPPLRLGSSLLSFTPRRSSVAPRALPALHPEFTLSPHLV.

We searched the NCBI database for homologs among available proteins using BLAST algorithm [14] but did not detect any significant similarities. The nucleotide context of the AUG codons also appeared to be suboptimal for translation initiation [15].

### 3.5. RNA Secondary Structure

**Figure 4:**
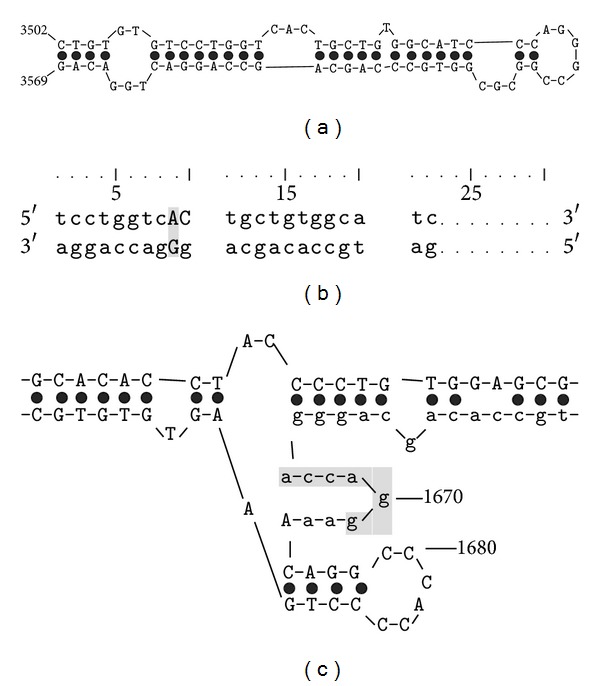
Transcribed locus Hs.633957 may code for a microRNA. (a) A hairpin-like fragment of the secondary structure was predicted for the BX119057 sequence by Mfold. Nucleotide positions are given according to the genome sequence starting from the leftmost TSS. (b) The potential interaction of the predicted mature miRNA with its target site in DPYS mRNA. (c) Fragment of the DPYS mRNA secondary structure. The target site for the miRNA is in lower case. The miRNA seed site-interacting region is shaded. Nucleotide positions are given according to the mRNA (acc. No. NM_001385.2).

To check the possibility that the putative gene under study codes for a microRNA we modelled secondary structure for the BX119057-like RNA isoform using Mfold software [8] and identified a 68 nt hairpin-like structure (Figure 4(a)). This hairpin was supported by 7 predicted secondary structure models with the lowest free energy (data not shown). It was formed by the nucleotides 3502–3569 of the putative gene and resembled hairpins of known pre-miRNAs. The 5′-strand of the hairpin stem holded a 22 nt fragment, which was complementary to a region of *DPYS* (dihydropyrimidinase) mRNA (Figure 4(b)). This 22 nt site was located in the coding region of the mRNA near the 3′ UTR. Interestingly, the DPYS mRNA region 1666–1671 complementary to the seed site of the potential miRNA is single stranded, which makes it easier to interact with the putative miRNA (Figure 4(c)). These data support the possibility that the transcribed locus Hs.633957 may code for a miRNA, and DPYS mRNA is its target.

### 3.6. Phylogenetic Analysis of the Gene

We used repeat-masked DNA sequence for transcribed region under study and 1 kb upstream region as a query to search NCBI databases for its homologs in available genomes using BLAST algorithm. Homology regions covering the whole gene were detected only in primates. They were all single copied. Two major conserved fragments were detected in nonprimate mammals. The first one was about 200 bp long and it was located about 500 bp upstream of the TSS. The second one was about 100 bp with its center located near the SD site. The overview of the gene conservation is presented in Supplementary Figure S6. It shows the “Vertebrate Multiz Alignment & Conservation (44 Species)” track of GB, which provides sequence conservation level within synthetic chromosome regions as calculated by PhastCons algorithm. In accordance with the data above, only short conserved regions can be observed.

**Figure 5:**
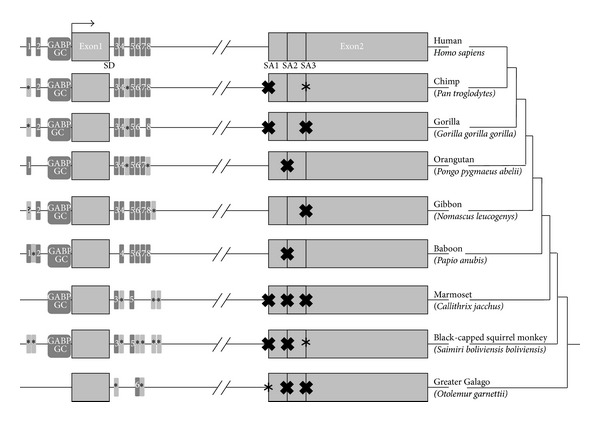
Emergence pattern of functional regions around ELFN1-AS1. The changes of the functional regions in the context of the phylogeny are shown. E-boxes are shown as grey boxes numbered 1–8. Lighter colour with “∗” sign indicates an alternative E-box sequence at the point. GABP and GC represent the GABP binding site and the GC-box. SD represents the splice donor site, SA1-SA3: the acceptor splice sites. Black crosses indicate the absence of a splice signal. The “∗” indicates presence of an alternative splice signal. Exons and introns are not to scale. Data for the nonprimate vertebrates are not shown.

Using the results of BLAST-search we extracted orthologous gene sequences from the most recent assemblies of primate genomes and made multiple alignment using ClustalW algorithm [16]. The alignment was then used to construct an average distance tree with Jalview v.2.7 software [17] (Supplementary Figure S7). The tree topology correlates with primate phylogeny [18]. Galagidae (*Otolemur garnettii*) forms an outgroup, indicating a distinct evolutionary pattern for the gene. We analysed the TSS region of the multiple alignment for the presence of core promoter elements corresponding to human counterparts. The GC-box [12] and GABP-binding [19] motifs were present in all primates, with an exception of *O. garnettii* (Figure 5). The SD site was intact in all primates, while the SA sites varied between species. Promoter region and the 5′-end of the intron were enriched with E-boxes in most of the primates.

## 4. Discussion

We have characterised *LOC100505644 uncharacterized LOC100505644 [Homo sapiens]* gene corresponding to the human transcribed locus Hs.633957 as a putative gene with tumor-specific expression by bioinformatic approach [1]. It was experimentally demonstrated to be transcribed in various tumors, but in normal tissues its expression seems to be weak and specific to liver, heart, and stomach [2–4]. Because transcription of the gene was tumor-related and very weak in normal tissues, we were unsure if it was functional. Alternatively, it could be a result of transcriptional noise. Thus, the status of this gene was uncertain. The data presented here confirm that *LOC100505644* is expressed mostly in tumors and indicate that it is a novel human gene. In accordance with Human Gene Nomenclature Committee regulations we propose to designate it *ELFN1-AS1*, ELFN1 antisense RNA 1 (non-protein coding).

We used an independent set of cDNA samples to recheck the expression of the gene in human tissues and tumors with PCR. We obtained results similar to our previous data [2–4], which show that *ELFN1-AS1* is a tumor-related gene (Figure 1). We used RACE and genetic database analysis to define the borders of the *ELFN1-AS1* transcript and identify its possible splice variants. Although a considerable diversity of transcript variants was predicted, only one splice variant predominated over others in healthy tissues and tumors, as defined by PCR. Such stability would not be expected for “junk” RNA. In fact, there was some variation in SA of the 1st intron (Figure 2), but the SD site was invariant, suggesting the presence of a strong splicing signal. The GT-repeat downstream of the SD site (Figure 3) is the primary candidate for this role, as GT/CA repeats are known to regulate activity of nearby splice sites [20, 21] and are widely spread in introns of human genes [22].

Knowing the TSS we could analyse the promoter region of the gene. We found that the TSS fell into a chromatin region rich in promoter- and enhancer-specific histone modifications. A GC-box, which is a common core promoter element [12], was identified right before the TSS in a DNaseI hypersensitive site, suggesting a core promoter responsive to Sp1 transcription factors family to be there. About 20 transcription factors were found to be specifically associated with the promoter region of the gene (Figures 3, S1–S5) in different cells; several of the DNA-binding TFs had putative binding sites within the region. Three cell lines—K-562, HeLa-S3, and GM12878—were best characterised in genetic databases in respect of TFs association with chromatin. Of those three, TFs binding to the promoter were observed in K-562 and HeLa-S3 cell lines (Figures 3, S1–S5). In agreement with this, *ELFN1-AS1* expression in K-562 cells was supported by data from Transcription Track of GB (Figure 3), and expression in HeLa-S3 cells was supported by 4 HeLa-S3-specific ESTs (GenBank acc. nos. AA190879, AA190847, AA488351, and AA488481). These data suggest that *ELFN1-AS1 *has its own promoter.

c-Myc/Max and PolII association with the chromosome region is of particular interest. c-Myc is one of the major regulators of cell cycle. Its upregulation in normal cells is associated with cell proliferation and protein synthesis [23]. c-Myc may act through the regulation of transcription pause release [24] or by attracting chromatin-modifying complexes in combination with Max [25]. We found 8 E-boxes in promoter proximal region and all of them rest in chromatin regions with which c-Myc and Max are associated in specific cells. Though the individual impact of each E-box into the gene regulation is a subject of further studies, the data presented here clearly indicate that *ELFN1-AS1* is a c-Myc-responsive gene.

Another noteworthy fact is the association of E2F family transcription factors with the *ELFN1-AS1* promoter. Transcription factors of E2F family regulate cell cycle through the genes involved in DNA synthesis [26]. They are also known to be able to work synergistically with Sp1 for gene activation [27]. Thus, regulation of the gene by E2Fs supports the idea that *ELFN1-AS1* must be normally active in proliferating cells. Since c-Myc overexpression is a common event for many malignancies [23] this may explain why we detect *ELFN1-AS1* expression in various kinds of tumors [2–4].

In general, the current data on TF association with chromatin, which is available from GB, is not sufficient to speculate about the details of *ELFN1-AS1* regulation, but it clearly indicates that *ELFN1-AS1* possesses its own promoter and is a subject of a complex regulation by various TFs, c-Myc/Max and E2F family proteins in particular. With regard to the tissue-specific transcription and reproducible RNA primary structure the human transcribed locus Hs.633957 may be considered “a complete chromosomal segment responsible for making a functional product,” which is a definition of a gene according to Snyder and Gerstein [28]. The function of this gene is yet to be identified; however, we consider it unlikely that *ELFN1-AS1* is translated and functions as a protein. Although there are 2 ORFs in *ELFN1-AS1* mRNA, neither of the corresponding proteins is present in protein databases. Also, the nucleotide context of the AUG codons [15] is suboptimal for translation initiation.

Based on the RNA secondary structure we speculate that *ELFN1-AS1* codes for a miRNA and its function is related to regulation of gene expression, with *DPYS* mRNA being its most probable target (Figure 4). This prediction is indirectly supported by the incomplete complementarity of the putative miRNA and its target site with unpaired nucleotides in position 9 and presence of adenine in the first position of the target site (Figure 4(c)). Such features are favourable for miRNAs action as supressors at RNA level [29, 30]. It was also supposed that the seed sites of the miRNAs serve as initiators for the miRNA-target interaction through fast target recognition [31]. In compliance with this idea, the putative miRNA seed site counterpart in the DPYS mRNA is single stranded (Figure 4(c)), while it is surrounded with double-stranded RNA regions. It is also known that DPYS participates in pyrimidine catabolism [32] and is active in liver and in solid tumors [33]. According to our data liver is one of the few normal tissues where weak expression of the gene is observed [2–4]. These data give indirect evidence in favour of the miRNA function of *ELFN1-AS1* and *DPYS* being its primary target. This is also in good correspondence with the idea that the gene is normally activated in proliferating cells. We are currently working on experimental verification of this prediction.

Another possibility is that *ELFN1-AS1* somehow interacts with *ELFN1* function, which is located on the opposite DNA strand. Elfn1 protein is selectively expressed by O-LM interneurons and directs the formation of highly facilitating pyramidal-O-LM synapses [34]. An antisense inside of *ELFN1* gene may also participate in regulation of this function; however, the only evidence of such a possibility so far is expression of *ELFN1-AS1* in fetal brain.

The origin of the gene is of particular interest. According to our data it originated from an intronic region of a conserved gene *ELFN1* (NCBI Ref. Seq. NM_001128636.2) in primate lineage. Nonprimate mammals had only small regions of homology with human *ELFN1-AS1*. Homologous sequences for the gene were identified in all primates, but DNA sequence from the representative of suborder Strepsirrhini *Otolemur garnettii* had more than 50% differences from its human counterpart and formed an outgroup on the phylogenetic tree. It was also different from other primates in that it had no GC-box, GABP, and E2F binding sites in the region, corresponding to human TSS (Figure 5). Thus, *ELFN1-AS1* could become transcriptionally active after the divergence of Strepsirrhini and Haplorhini primates. It is noteworthy that all the Haplorhini primates had a region with 5 or more E-boxes downstream of the DS site. This indicates that this gene could always be c-Myc-responsive. This gene combines features of predominant expression in tumors and evolutionary novelty.

## 5. Conclusion

Here we describe a novel human non-protein coding gene *ELFN1-AS1*. We give a description of its splice forms and major regulatory elements and present evidence that it may code for a miRNA. We also show that this gene originated in primates *de novo*. Considering the tumor-related expression of the gene, we suppose that it might be a regulatory gene playing some important role in carcinogenesis. The further detailed studies of its function and regulation would be interesting.

## Supplementary Material

Supplementary figures 1-5 show the location of predicted transcription factors binding sites within the promoter region of the ELFN1-AS1 gene and binding of transcription factors, as defined by analysis of Chip-Seq data that is available from UCSC Genome Browser. Supplementary Figure 6 provides the overview of gene conservation. Supplementary Figure 7 shows the average distance tree based on ELFN1-AS1. Supplementary File 1 shows the sequence of the promoter region of the gene and location of the predicted transcription factors binding sites.

## Figures and Tables

**Table 1:** Gene-specific amplification primers.

No.	Primer name	Primer sequence
1	5gsp1	GCTGAGAGTGAATTCGGGGTGCAG
2	5gsp1n	GCTGCTGCGTCTCGGAGTGAATG
3	5gsp2	GACTGGGAGTGAGAAGGAGC
4	5gsp2n	GGAGTGAGAAGGAGCGGAG
5	5gsp3	GGTCTTTACTCCCATTCAACGGAAGAG
6	5gsp3n	CATTCAACGGAAGAGGAAGCAGAGC
7	3gsp1	CCTCCTGTCATTCACTCCGAGACGC
8	3gsp1n	CATTCACTCCGAGACGCAGCAGC
9	Upstream primer (UP)	GTGGCGCCTCAGCCACAATC
10	Nested upstream primer (NUP)	GCCTCAGCCACAATCGTAAT
11	Distal downstream primer (DDP)	GTGAGAAACCACAAGCTCCCTG
12	Nested distal downstream primer (NDDP)	GGTCTTTACTCCCATTCAA
13	Proximal downstream primer (PDP)	CAGGTTCTTCAGCCAGGAAG
14	Nested proximal downstream primer (NPDP)	GGAGTGAGAAGGAGCGGAG
