# Supplementary material for: ELFN1-AS1: A Novel Primate Gene with Possible MicroRNA Function Expressed Predominantly in Human Tumors
Source: Biomed Res Int. 2014 Feb 24;2014:398097. doi: 10.1155/2014/398097 (PMC3953637; doi:10.1155/2014/398097)
Supplement: Supplementary file 1 — Supplementary figures 1-5 show the location of predicted transcription factors binding sites within the promoter region of the ELFN1-AS1 gene and binding of transcription factors, as defined by analysis of Chip-Seq data that is available from UCSC Genome Browser. Supplementary Figure 6 provides the overview of gene conservation. Supplementary Figure 7 shows the average distance tree based on ELFN1-AS1. Supplementary File 1 shows the sequence of the promoter region of the gene and location of the predicted transcription factors binding sites. [file 398097.f1.zip › Supplementary_file_1.pdf]

```
                                                                  YY1/1
-1000     -990      -980      -970      -960      -950      -940      -930
  |....|....|....|....|....|....|....|....|....|....|....|....|....|....|....|....
  gctctattcctcagcctccactggaccctgagggtctccaggcaggacggtgcctcctccatctgcctagtgtctgctga
                   E2F/1             E2F/2
-920      -910      -900      -890      -880      -870      -860      -850
  |....|....|....|....|....|....|....|....|....|....|....|....|....|....|....|....
  gcctggacacagaggatgtggcgaccgagctgtaggcggggaagccccaggcaggaaggaccctgcagacggggctggag
                        E2F/3                 YY1/2 YY1/3
-840      -830      -820      -810      -800      -790      -780      -770
  |....|....|....|....|....|....|....|....|....|....|....|....|....|....|....|....
  gtacgtccggggagcgaggcatcccgctgggctcctgctccccatggacccccccaccactaccctcgagataaggggg
                           E2F/4                                YY1/4
-760      -750      -740      -730      -720      -710      -700      -690
  |....|....|....|....|....|....|....|....|....|....|....|....|....|....|....|....
  cccctggtgtgcgacctcacctcccggccgcctgtgttttccctgcaggatagagaaatggggtgtgtttgcttgttaata
                                          E2F/5
-680      -670      -660      -650      -640      -630      -620      -610
  |....|....|....|....|....|....|....|....|....|....|....|....|....|....|....|....
  tgtggatttacagtcggcctctgtcaccgagccgtggcctggcgggcagccagctgggtgtatttcactgatattgacgt
                        E2F/6                                     YY1/5
-600      -590      -580      -570      -560      -550      -540      -530
  |....|....|....|....|....|....|....|....|....|....|....|....|....|....|....|....
  ttgcaactagtctgcactgaagcggtttaaattagagaaaaaaaatataatgatgtaaaatggggtttttcctccctttg

-520      -510      -500      -490      -480      -470      -460      -450
  |....|....|....|....|....|....|....|....|....|....|....|....|....|....|....|....
  gaaacttcataaagaacaattttgttccggctcctggagaagagtctatttctgatttaaagagagggagaagtcagacg
  YY1/6                     E2F/7                                           E2F/8
-440      -430      -420      -410      -400      -390      -380      -370
  |....|....|....|....|....|....|....|....|....|....|....|....|....|....|....|....
  gcatggggctacaacgaggtcctagcgggcctcggggagctaacccaggcttggcctggagtggccaggggacgccaa
                                      YY1/7                    YY1/8     E-box/1
-360      -350      -340      -330      -320      -310      -300      -290
  |....|....|....|....|....|....|....|....|....|....|....|....|....|....|....|....
  gcccagccgggcaggacctggctaggcccactcaaaaatggctcaggggggctgcttgggtagggaaaatggggtccccac
                            YY1/9                       YY1/10
-280      -270      -260      -250      -240      -230      -220      -210
  |....|....|....|....|....|....|....|....|....|....|....|....|....|....|....|....
  gtgggtccggttggatgtgaaatccaccatcacgggagtcagcgctgtcttggtcaccatcatgccgtgtgcacctgccag
                       YY1/11        YY1/12
-200      -190      -180      -170      -160      -150      -140      -130
  |....|....|....|....|....|....|....|....|....|....|....|....|....|....|....|....
  ctcgcacccacgccagcccctccatccctccccaccatccctgcaccgtgcccagacaacctgtgccaatgctgggattt
                                          E-box/2             E2F/9          YY1/13E2F/10
-120      -110      -100      -90       -80       -70       -60       -50
  |....|....|....|....|....|....|....|....|....|....|....|....|....|....|....|....
  agctttcccaagcctcctgggggcccaggtctcaggcagaggcgcgtgctggccggggcgggccaagggcatgggcgg
  YY1/14 E2F/10                    GABP  GC-box  E2F/11 E2F/12
 -40       -30       -20       -10       +1        10        20        30
  |....|....|....|....|....|....|....|....|....|....|....|....|....|....|....|....
  atggcgtccggctgtgcccggcctccggaagggcgggtctGGCCAGCGGCCGCACAGGAAGCGTGTAGGAAGCGTGGCG

  40        50        60        70        80        90        100       110
  |....|....|....|....|....|....|....|....|....|....|....|....|....|....|....|....
  CCTCAGCCACAATCGTAATCACCTTTAATCTCTTGCTCAAAATAACCCAAAGTCAAGgtaaggaggattacagggcctaa
                                                         E2F/13
 120       130       140       150       160       170       180       190
  |....|....|....|....|....|....|....|....|....|....|....|....|....|....|....|....
  gagccctcaccacaggcacagggaggaggcttgggggcctggggctgctccggctctcctgggcccgcagctgagggctg
          E-box/3YY1/15 E-box/4     E-box/5       E-box/6
 200       210       220       230       240       250       260       270
  |....|....|....|....|....|....|....|....|....|....|....|....|....|....|....|....
  gagggctgcatgcacatggggttcacgtgtgtgtgtgtgcatgtgtgtttgtggacacatgtatacctgtgtccacccac
  E-box/7  E-box/8
 280       290       300       310       320       330       340       350
  |....|....|....|....|....|....|....|....|....|....|....|....|....|....|....|....
  acacgtgtttgtacacgtgagccctcgtatgtgtgtgccaggttgtctgtgtgtgtgtacagatgtgtgtgcacaggtgt
                                      YY1/16
 360       370       380       390       400       410       420       430
  |....|....|....|....|....|....|....|....|....|....|....|....|....|....|....|....
  gtacacagggcaggtgattgtgtacatgagtgcgcaggccatgctcactggggccctgggcagagcaggtgtcactgagg

 440       450       460       470       480       490       500
  |....|....|....|....|....|....|....|....|....|....|....|
  acccccctgtctgggcagcgaggcctggggctctggtgcgtctggggctgggtggaggcaggt
```
