# Supplementary figures and images for: ELFN1-AS1: A Novel Primate Gene with Possible MicroRNA Function Expressed Predominantly in Human Tumors
Source: Biomed Res Int. 2014 Feb 24;2014:398097. doi: 10.1155/2014/398097 (PMC3953637; doi:10.1155/2014/398097)

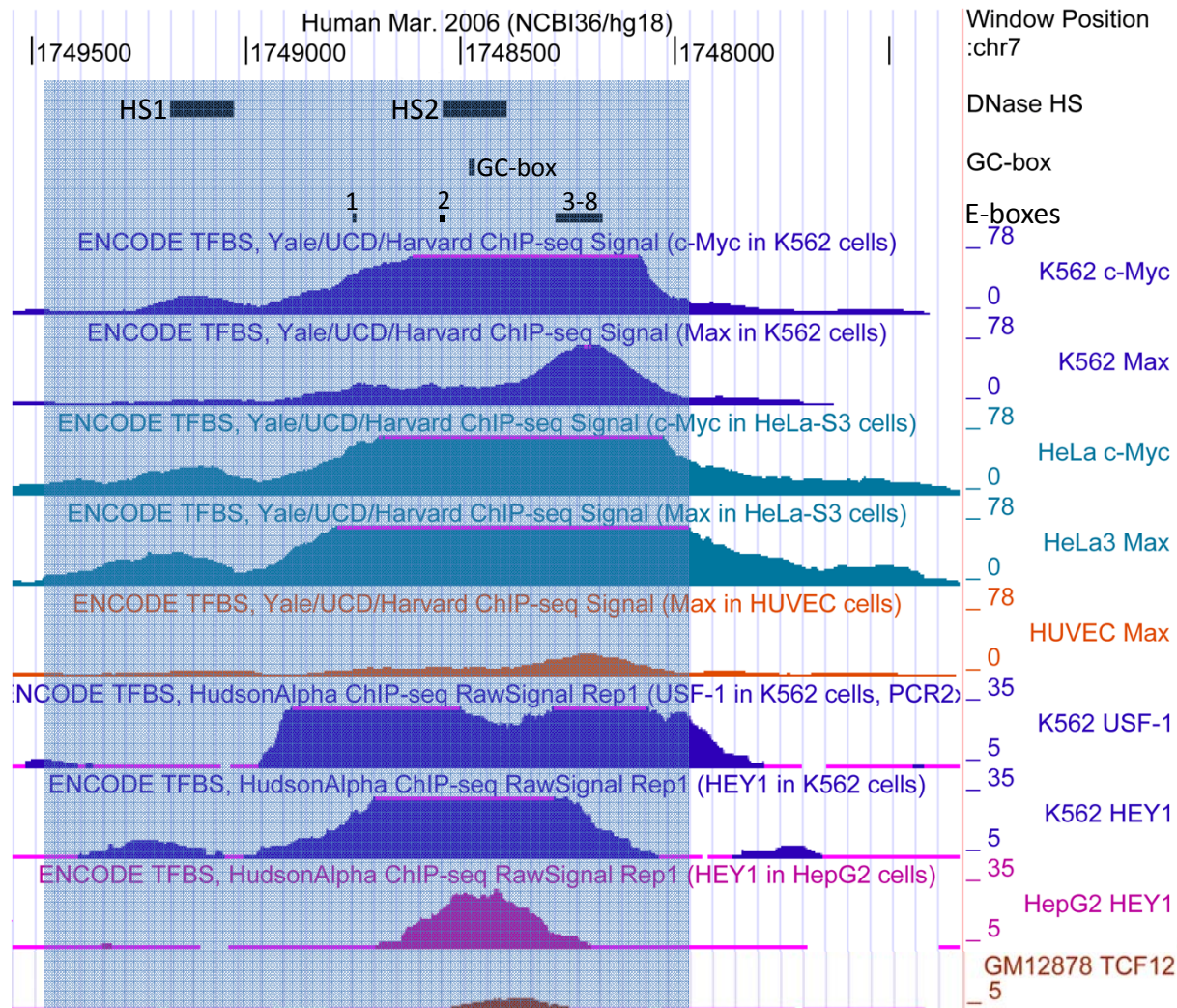

Supplement: Supplementary file 1 — Supplementary figures 1-5 show the location of predicted transcription factors binding sites within the promoter region of the ELFN1-AS1 gene and binding of transcription factors, as defined by analysis of Chip-Seq data that is available from UCSC Genome Browser. Supplementary Figure 6 provides the overview of gene conservation. Supplementary Figure 7 shows the average distance tree based on ELFN1-AS1. Supplementary File 1 shows the sequence of the promoter region of the gene and location of the predicted transcription factors binding sites. [file 398097.f1.zip › Supplementary_figure1.pdf]

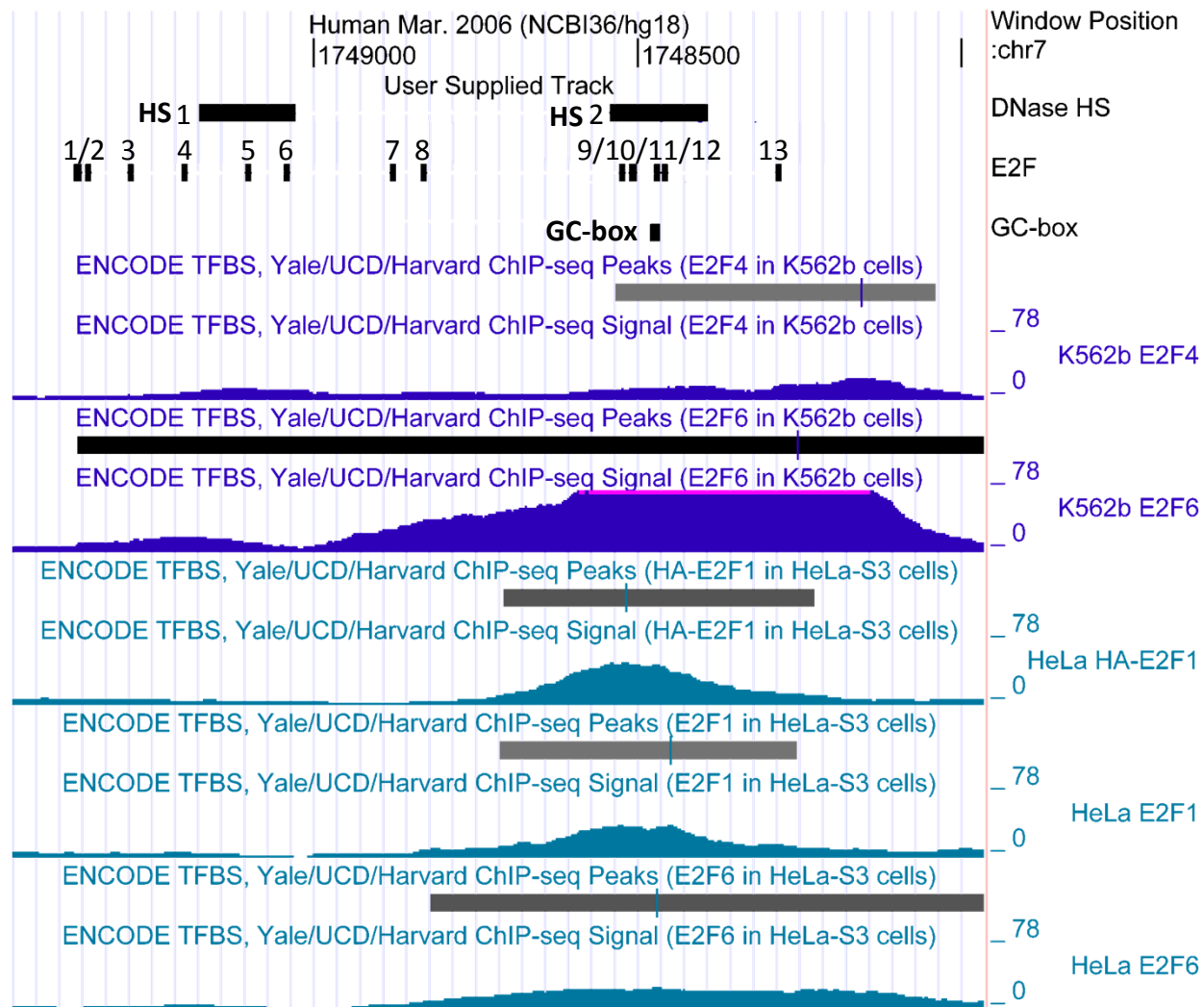

Supplement: Supplementary file 1 — Supplementary figures 1-5 show the location of predicted transcription factors binding sites within the promoter region of the ELFN1-AS1 gene and binding of transcription factors, as defined by analysis of Chip-Seq data that is available from UCSC Genome Browser. Supplementary Figure 6 provides the overview of gene conservation. Supplementary Figure 7 shows the average distance tree based on ELFN1-AS1. Supplementary File 1 shows the sequence of the promoter region of the gene and location of the predicted transcription factors binding sites. [file 398097.f1.zip › Supplementary_figure2.pdf]

# YY1

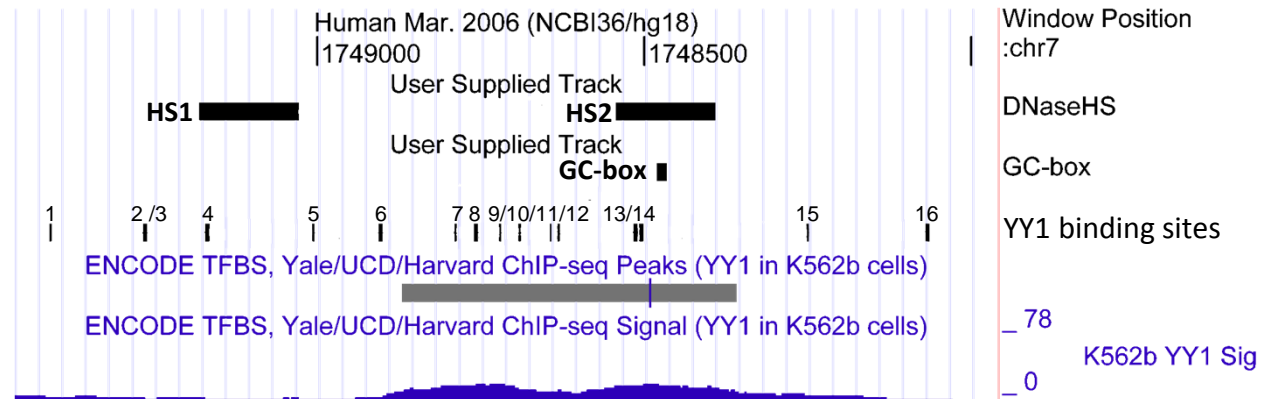

Supplement: Supplementary file 1 — Supplementary figures 1-5 show the location of predicted transcription factors binding sites within the promoter region of the ELFN1-AS1 gene and binding of transcription factors, as defined by analysis of Chip-Seq data that is available from UCSC Genome Browser. Supplementary Figure 6 provides the overview of gene conservation. Supplementary Figure 7 shows the average distance tree based on ELFN1-AS1. Supplementary File 1 shows the sequence of the promoter region of the gene and location of the predicted transcription factors binding sites. [file 398097.f1.zip › Supplementary_figure3.pdf]

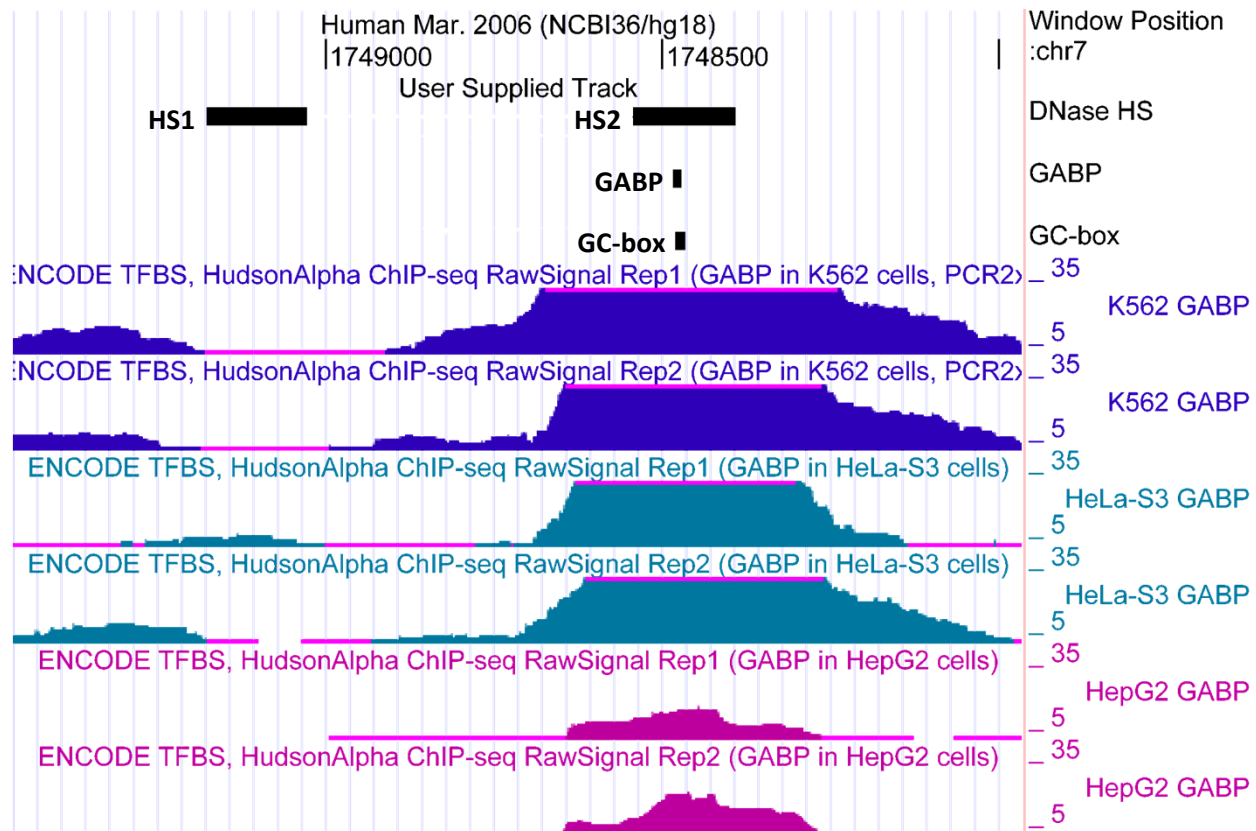

Supplement: Supplementary file 1 — Supplementary figures 1-5 show the location of predicted transcription factors binding sites within the promoter region of the ELFN1-AS1 gene and binding of transcription factors, as defined by analysis of Chip-Seq data that is available from UCSC Genome Browser. Supplementary Figure 6 provides the overview of gene conservation. Supplementary Figure 7 shows the average distance tree based on ELFN1-AS1. Supplementary File 1 shows the sequence of the promoter region of the gene and location of the predicted transcription factors binding sites. [file 398097.f1.zip › Supplementary_figure4.pdf]

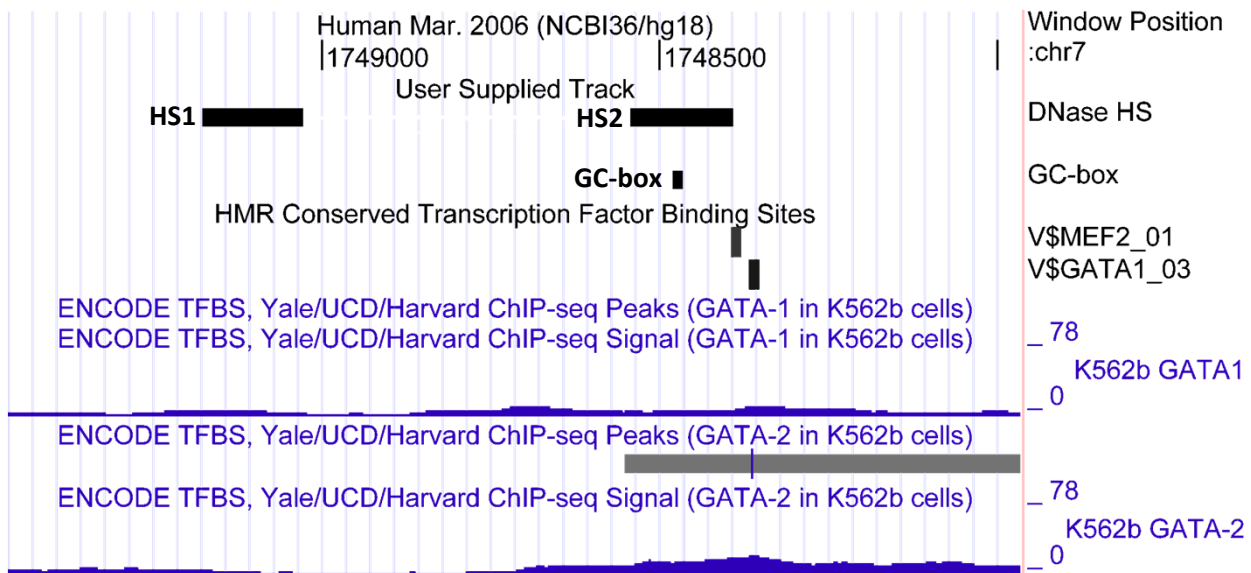

Supplement: Supplementary file 1 — Supplementary figures 1-5 show the location of predicted transcription factors binding sites within the promoter region of the ELFN1-AS1 gene and binding of transcription factors, as defined by analysis of Chip-Seq data that is available from UCSC Genome Browser. Supplementary Figure 6 provides the overview of gene conservation. Supplementary Figure 7 shows the average distance tree based on ELFN1-AS1. Supplementary File 1 shows the sequence of the promoter region of the gene and location of the predicted transcription factors binding sites. [file 398097.f1.zip › Supplementary_figure5.pdf]

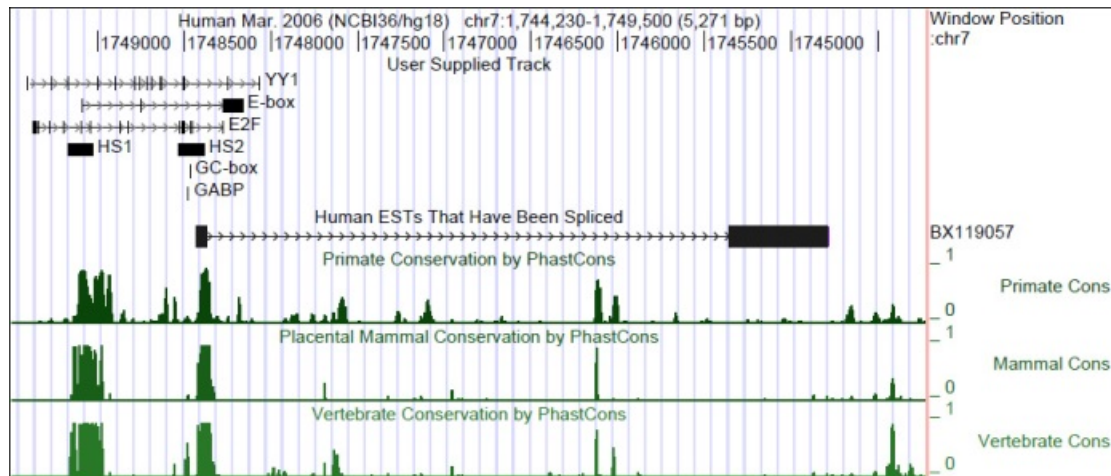

Supplement: Supplementary file 1 — Supplementary figures 1-5 show the location of predicted transcription factors binding sites within the promoter region of the ELFN1-AS1 gene and binding of transcription factors, as defined by analysis of Chip-Seq data that is available from UCSC Genome Browser. Supplementary Figure 6 provides the overview of gene conservation. Supplementary Figure 7 shows the average distance tree based on ELFN1-AS1. Supplementary File 1 shows the sequence of the promoter region of the gene and location of the predicted transcription factors binding sites. [file 398097.f1.zip › Supplementary_figure6.pdf]

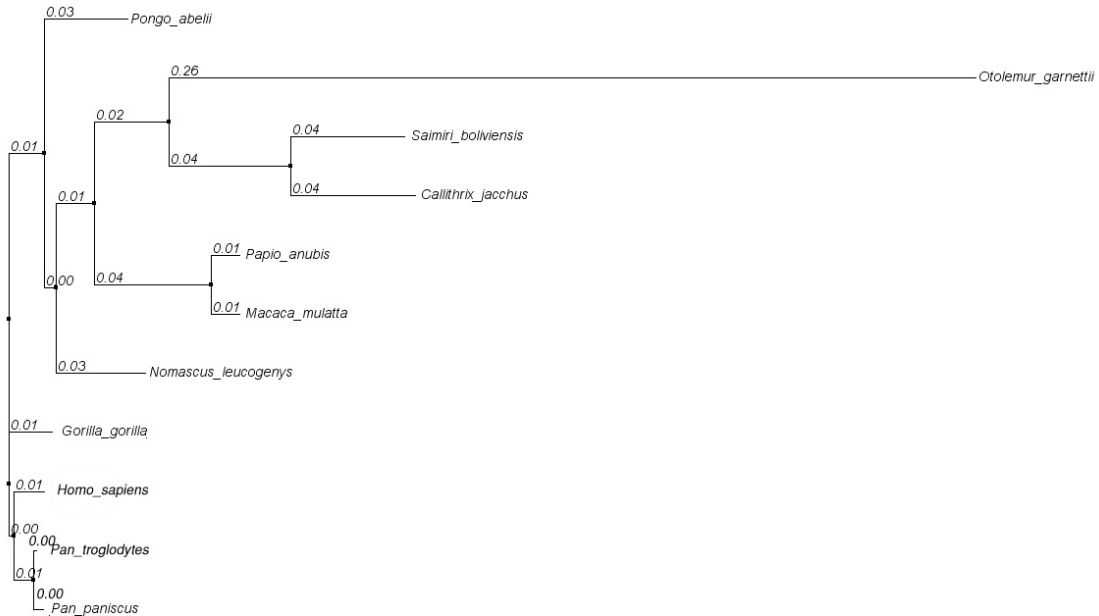

Supplement: Supplementary file 1 — Supplementary figures 1-5 show the location of predicted transcription factors binding sites within the promoter region of the ELFN1-AS1 gene and binding of transcription factors, as defined by analysis of Chip-Seq data that is available from UCSC Genome Browser. Supplementary Figure 6 provides the overview of gene conservation. Supplementary Figure 7 shows the average distance tree based on ELFN1-AS1. Supplementary File 1 shows the sequence of the promoter region of the gene and location of the predicted transcription factors binding sites. [file 398097.f1.zip › Supplementary_figure7.pdf]
